# Mesenchymal Stem Cell Therapy Overcomes Steroid Resistance in Severe Gastrointestinal Acute Graft-Versus-Host Disease

**DOI:** 10.1155/2019/7890673

**Published:** 2019-05-21

**Authors:** Kyoko Moritani, Reiji Miyawaki, Kiriko Tokuda, Fumihiro Ochi, Minenori Eguchi-Ishimae, Hisamichi Tauchi, Mariko Eguchi, Eiichi Ishii, Kozo Nagai

**Affiliations:** ^1^Department of Pediatrics, Ehime University Graduate School of Medicine, Ehime, Japan; ^2^Department of Pediatrics, Ehime Prefectural Central Hospital, Ehime, Japan

## Abstract

The authors describe the high effectiveness of human mesenchymal stem cell (hMSC) therapy to treat steroid-refractory gastrointestinal acute graft-versus-host Disease (aGVHD) in a 15-year-old boy with acute lymphoblastic leukemia (ALL). He received allogeneic hematopoietic stem cell transplantation due to high-risk hypodiploid ALL. Around the time of engraftment, he developed severe diarrhea following high-grade fever and erythema. Although methylprednisolone pulse therapy was added to tacrolimus and mycophenolate mofetil, diarrhea progressed up to 5000~6000 ml/day and brought about hypocalcemia, hypoalbuminemia, and edema. Daily fresh frozen plasma (FFP), albumin, and calcium replacements were required to maintain blood circulation. After aGVHD was confirmed by colonoscopic biopsy, MSC therapy was administered. The patient received 8 biweekly intravenous infusions of 2×10^6^ hMSCs/kg for 4 weeks, after which additional 4 weekly infusions were performed. A few weeks after initiation, diarrhea gradually resolved, and at the eighth dose of hMSC, lab data improved without replacements. MSC therapy successfully treated steroid-refractory gastrointestinal GVHD without complications. Despite life-threatening diarrhea, the regeneration potential of children and adolescents undergoing SMC therapy successfully supports restoration of gastrointestinal damage. Even with its high treatment costs, SMC therapy should be proactively considered in cases where young patients suffer from severe gastrointestinal GVHD.

## 1. Introduction

Allogenic hematopoietic stem cell transplant (Allo-HSCT) improves outcomes of high-risk hematological malignancy and severe non-malignant hematological diseases [1, 2]. Despite donor human leucocyte antigen (HLA) typing method and optimization of donor selection, HLA-mismatched transplantations are unavoidable due to a shortage of donors. Even though posttransplantation supportive care has improved, acute graft-versus-host disease (aGVHD) remains a leading cause of both transplantation-related mortality (TRM) and morbidity following allogeneic HSCT. Steroids, often added to methotrexate and calcineurin inhibitors, are employed as the first-line treatment for aGVHD; however, in 30–50% of patients, aGVHD is not controlled with first-line steroid therapy [3]. Steroid-refractory GVHD outcomes are dismal, so further therapeutic intervention inhibitors are required. Second-line agents, including antithymocyte globulin (ATG), mycophenolate mofetil (MMF), and infliximab, provide limited improvement owing to a higher risk of infectious complications, immunosuppression-related toxicity, and incomplete GVHD remission [4–6].

Bone marrow contains pluripotent mesenchymal stem cells (MSCs) that form bone, cartilage, adipose tissue, and muscle. These stem cells are not immunogenic and escape recognition by allo-reactive T cells and natural killer cells. Mesenchymal stem cells given intravenously have been well tolerated [7]. Preliminary reports of co-transplantation of MSCs and HSCs from HLA-identical siblings show a reduction in acute and chronic GVHD [8]. In 2004, Le Blanc and coworkers first demonstrated that haploidentical MSC infusion dramatically improved severe steroid-refractory aGVHD in a pediatric patient with acute lymphoblastic leukemia (ALL) [9]. A further phase II study of MSC therapy for severe steroid-refractory GVHD was conducted by same Le Blanc team. Among 55 patients, including Grade III ~ IV at 90%, 30 patients had a complete response and 9 showed improvement. Complete responders had lower transplantation-related mortality and a higher survival rate than did patients with partial or no response [10]. Following that, smaller and larger studies investigating the effect of MSCs in steroid-refractory GVHD were conducted, and its safety and effectiveness have been shown [11–13]. Nowadays, in many countries, hMSC infusion is approved as a second-line therapy for steroid-refractory GVHD.

The gastrointestinal tract is highly affected by preconditioning treatments. In the earliest pathophysiological events of GVHD, neoangiogenesis [14] and infiltration of innate neutrophil granulocytes and monocytes are caused [15, 16]. Recipient neutrophils induce epithelial cells damage and increased intestinal permeability through their activation and production of reactive oxygen species in gastrointestinal tract [15]. Bacteria, fungi, and viruses infiltrate to submucosa and activate antigen presenting cells (APCs) through toll-like receptor (TLR) and nucleotide-binding oligomerization domein-like receptor (NOD-R). Damaged tissue and activated APCs induce various immune cell alloreactions, especially donor T cells, resulting in GVHD [17]. Lower gastrointestinal tract GVHD is the predominant cause of morbidity and mortality from allo-HSCT. This complication has poor outcomes, with 25% overall survival [18].

In this report, we describe a case of MSC therapy improving life-threatening diarrhea caused by gastrointestinal GVHD refractory to continuous steroid and pulse therapy. This case report suggests MSC therapy to be highly effective for child and adolescent patients with severe steroid-refractory gastrointestinal GVHD.

## 2. Case Report

A 14-year-old boy was referred to a previous hospital with intermittent fever and joint pain. Laboratory findings revealed inflammatory change (C-reactive protein [CRP], 12.91mg/dL; ferritin, 246 ng/mL; soluble IL-2 receptor [sIL2R], 1389U/mL), normal white blood cell (WBC) count, 6880/*μ*L, with 2% lymphoblasts, moderate thrombocytopenia (platelet [PLT] was 6.4 x 10^4^/*μ*L), normal transaminase levels, high lactate dehydrogenase (LDH), 1315U/L, and slightly abnormal blood coagulation test. Bone marrow aspiration showed that 56.2% of nucleated cells were lymphoblasts with immature nuclei, high N/C ratio, and positive staining for PAS. Flow cytometry revealed positivities for CD19, CD20, CD22, c-CD79, CD38, CD99 and HLA-DR, and a weak positivity for CD10. Although gene rearrangement, which frequently occurs in ALL, was not detected, low-hypodiploid with 36 or 37 chromosomes was detected in a chromosome test. Based on these findings, the diagnosis of B-lymphoblastic lymphoma (BLL) with hypodiploid was made. The patient was judged to have high-risk ALL and was scheduled to receive multidrug chemotherapy followed by high-dose chemotherapy with allo-HSCT.

**Figure 1:**
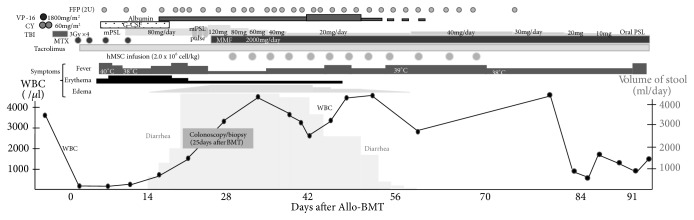
*Clinical course of patient.* VP-16: etoposide; CY: cyclophosphamide; TBI: total body irradiation; MTX: methotrexate; G-CSF: granular-colony stimulating factor; mPSL: methylprednisolone; PSL: predonisolone; MMF: mycophenolate mofetil.

**Figure 2:**
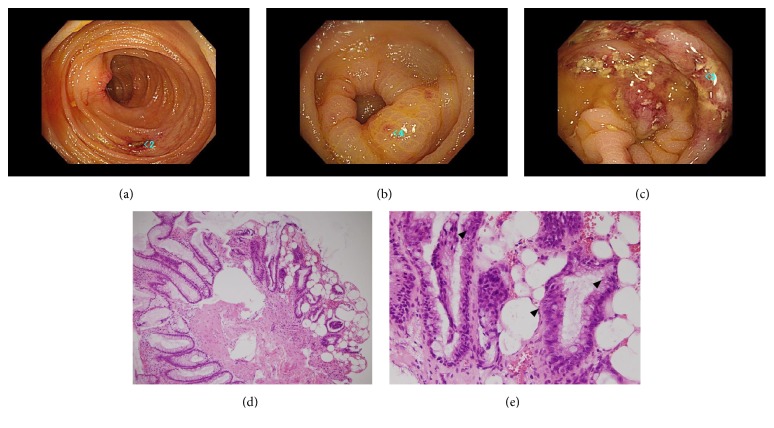
*Colonoscopy image at ascending colon (a), at sigmoid colon (b), and at rectum (c). H-E staining x100 of biopsy specimen (d). Apoptotic bodies (arrows) at x400 (e).* Edematous surface and scattered erosion were observed at whole colon and rectum. (a~c) Desquamated epithelium, interstitial edema, and submucosal fibrosis were seen as a result of inflammation. (d) Enlarged image showed submucosal lymphocyte infiltration and apoptotic bodies. There were no inclusion body cells (e).

Multidrug chemotherapy according to the JPLSG ALL-B12 protocol, which is BFM-based, consisting of steroid, Vincristine, anthracyclines, and L-asparagenase [19], was administered to the patient. After induction chemotherapy, he attained a complete clinical remission on day 33 after initiation. During intensification courses, minimal residual disease-polymeric chain reaction (MRD-PCR) targeting immunoglobulin heavy chain (IgH) in bone marrow was not detected. The patient was transferred to our hospital and underwent allogeneic bone marrow transplantation (BMT) with a conditioning regimen including 12 Gy total body irradiation, etoposide, and cyclophosphamide. A donor mismatched for two HLA antigens, -C and -DR, was selected due to a small number of candidates. Tacrolimus and short-term methotrexate (MTX), 15mg/m^2^ on day 1 and 10mg/m^2^ on days 3, 6, and 11, were administered as basic GVHD prophylaxes. The clinical course summary after HSCT is shown in Figure 1. Seven days after transplantation, engraft syndrome induced high-grade fever and progressive systemic erythema (Stage 3). G-CSF, 5*μ*g/kg/day of Lenograstim was administered from day 5 but was stopped on day 17 to prevent immune reaction overload. Erythema and fever showed no improvement, and CRP rose to 20.2mg/dL at peak value. Therefore, 1.33mg/kg/day (80mg) of methylprednisolone was added to tacrolimus and administered to the patient from day 7, improving the immune reactive symptoms. However, watery diarrhea appeared and gradually increased up to 5000~6000ml/day. Engraftment of neutrophil cells (neutrophil count ≧ 500/*μ*L for 3 days) was observed 14 days after transplant. Cytomegalovirus (CMV) DNA in blood was consistently negative. A methylprednisolone pulse from day 18 to 20 was followed by the administration of 2mg/kg of mPSL and MMF with dietary restrictions on day 21, but the watery diarrhea did not improve. Severe hypoalbuminemia (1.6~2.0 g/dL) progressed due to intestinal tract leaking, and replacement of FFP and albumin on consecutive days was unavoidable in order to maintain blood circulation. Severe hypocalcemia was observed, and an intensive supplement of calcium preparation was also required. Colonoscopy showed edematous surface and scattered erosion from ascending colon to rectum, colonoscopic biopsy revealed desquamated epithelium, interstitial edema, disordered structure of ducts, and submucosal fibrosis. Apoptosis with nuclear dust was observed occasionally in epithelium as a feature of gastrointestinal GVHD. There were no cells with inclusion body formation (Figure 2). After acute gastrointestinal GVHD was confirmed, MSC therapy at a dose of 2×10^6^ hMSCs/kg twice per week was added to daily methyl prednisolone treatment on day 28. To avoid steroid-related side effects, mPSL was gradually tapered: 80mg from day 26 to 29, 60mg from day 30 to 33, 40mg from day 34 to 37, and 20mg from day 38. At the fifth dose of hMSC, the volume of diarrhea decreased to around 1500ml/day, and eventually to 200ml/day, with stool form at the eighth dose. An additional 4 doses were administered weekly following the twice-per-week induction dose, and MSC therapy was finished after a total of 12 doses. Albumin and calcium concentrations were easily maintained without replacement. Dietary restrictions were gradually removed, and methyl prednisolone was successfully tapered without relapse of intestinal symptoms. At day 60, the mPSL dose was increased to 40mg per day due to fever caused by GVHD. The fever had a good response to steroids and was transient, allowing for a smooth tapering of mPSL to 5mg of oral prednisolone. A chromosome test of bone marrow (BM) cells on day 88 revealed the complete replacement of the BM cells by female type (donor type, 46,XX). At 130 days after transplant, the patient was discharged. Although the patient requires a low dose of steroid for the treatment of appetite loss and gastrointestinal discomfort, he is in stable condition and has been without disease relapse at one year after transplant.

## 3. Discussion

In this report, the patient suffered from engraft syndrome followed by lower gastrointestinal aGVHD. The diagnosis of causes leading to posttransplant diarrhea is a complex process. The differential diagnosis with infection including CMV, adenovirus, rotavirus, norovirus, and Clostridium difficile, is a critical point before conducting immunosuppression treatment for gastrointestinal GVHD [20]. The possibility of intestinal tract infection was excluded by analyzing stool specimen and culture, determining the presence of CMV DNA in serum, observation and biopsy under colonoscopy. Although the patient had steroid-refractory progression and life-threatening diarrhea, MSC therapy overcame steroid-refractory lower intestinal GVHD.

In a multicenter study of hMSC therapy for 55 cases with steroid-refractory GVHD (30 adult cases and 25 child cases), the overall response rate (RR) was 70.9%. Child cases had a better RR to MSC therapy (84.0%) compared to adult cases (70.9%) [10]. The effectiveness of hMSC therapy for aGVHD, including gastrointestinal GVHD, has been demonstrated in a phase II study. Twenty-five cases with steroid-refractory GVHD (grade III: 22 cases, grade IV: 3 cases) were enrolled. Results showed that at 24 weeks after initiation of MSC therapy, 12 cases (48%) remained in CR of GVHD for 28 days. The survival was significantly better in patients showing overall response (OR; CR+PR) than in those showing no OR at 4 weeks [21]. In multicenter Phase III study, Prochymal® (n = 163) or placebo (n = 61) were added to standard care in 244 patients with steroid-refractory GVHD (skin involvement n =144, gastrointestinal involvement n = 179, liver involvement n =61). Prochymal provided better response rate vs placebo at day 100 to liver (76% vs 47%) and gut (82% vs 68%) compared to skin involvement (78% vs 77%) [22]. Ball LM et al conducted a clinical study of hMSC therapy in a cohort of 37 children with steroid-refractory grade III-IV aGVHD. CR was observed in 24 children (65%) and PR in 8 children. Among 22 children with gastrointestinal GVHD, effective response was observed with CR in 19 children, and resolution of gastrointestinal symptoms occurred at a median time of 11 days [23]. Another study of hMSC treatment for gastrointestinal GVHD in pediatric patients demonstrated favorable results. Although all 12 patients had grade III-IV gastrointestinal GVHD even after multiple immunosuppression therapies, complete resolution of gastrointestinal symptoms occurred in 9 (75%) patients. Clinical responses, particularly in the gastrointestinal system, were seen in the majority of children with severe refractory aGVHD [24].

Tissue injury following high-dose chemotherapy and total body irradiation used in preparation for HCT triggers GVHD. Tissue damage, affecting primarily the gastrointestinal tract, generates a robust inflammatory response with release of cytokines that facilitate antigen recognition by allo-reactive donor T cells [17]. Human and animal models demonstrated that MSCs migrate specifically to damaged tissue sites exhibiting inflammation, although most become trapped in the microvasculature of the lung [25, 26]. T helper (Th) 1/Th2 balance has been identified as significant in GVHD [27]. MSCs interact with T cells and induce a Th1 to Th2 shift. Th1 cells reduce interferon-*γ*, and Th2 cells increase secretion of interleukin (IL)-4 induced through the interaction with human MSCs [28]. Th17 cells, CD4 T cells producing proinflammatory cytokine, have been recognized as playing a pivotal role in aGVHD. MSCs prevent Th cells from differentiation into Th17 subset through PGE2 production [29]. MSCs are shown to have the ability to repair damaged tissue and differentiate into the cells of that tissue [30]. A clinical trial was conducted to repair damaged tissue associated with HSCT or aGVHD using hMSCs [31].

Once atrophy and desquamation in colon epithelium are caused by inflammation of GVHD, restoration of gut epithelium following immunomodulation takes several weeks. Therefore, the patient had persistent diarrhea and increased stool frequency up to 21 days after initiation of MSCs. Prompt analyzing of stool and serum plus endoscopy for excluding infectious disease are required to conduct MSC therapy in adequate timing.

While hMSC therapy for severe steroid-refractory GVHD is approved for coverage by the national health insurance system in Japan, the therapy itself is quite expensive, and physicians should take care when selecting cases to receive MSC therapy. In our experience, MSC therapy should be strongly considered for administration in pediatric patients with severe gastrointestinal GVHD, even after steroid pulse therapy.

In conclusion, we had an excellent outcome when treating a steroid-refractory gastrointestinal GVHD patient using hMSC therapy. Even though the patient had life-threatening watery diarrhea, steroids were successfully tapered without a recurrence of symptoms. The immunomodulation and regeneration induced by MSC therapy might be more effective for the pediatric population because of their rapid tissue restoration. When encountering a pediatric patient with severe gastrointestinal GVHD resistant to steroid pulse therapy, MSC therapy administration should be given active consideration following stool-serum test and endoscopy to exclude the possibility of infection.
